# Widening of Dynamic Detection Range in Real-Time Angular-Interrogation Surface Plasmon Resonance Biosensor Based on Anisotropic Van Der Waals Heterojunction

**DOI:** 10.3390/bios14120601

**Published:** 2024-12-08

**Authors:** Xiantong Yu, Jing Ouyang, Zhao Li, Chaojun Shi, Longfei Wang, Jun Zhou, Min Chang

**Affiliations:** Key Laboratory of Optical Technology and Instrument for Medicine, Ministry of Education, College of Optical-Electrical and Computer Engineering, University of Shanghai for Science and Technology, Shanghai 200093, China

**Keywords:** angular-interrogation surface plasmon resonance (SPR) sensor, dynamic detection range, two-dimensional (2D) van der Waals heterojunction (vdWhs), black phosphorus (BP)

## Abstract

Surface plasmon resonance (SPR) biosensors have experienced rapid development in recent years and have been widely applied in various fields. Angular-interrogation SPR biosensors play an important role in the field of biological detection due to their advantages of reliable results and high stability. However, angular-interrogation SPR biosensors also suffer from low detection sensitivity, poor real-time performance, and limited dynamic detection range, which seriously restricts their application and promotion. Therefore, we designed an angular-interrogation SPR biosensor based on black phosphorus (BP)/graphene two-dimensional (2D) van der Waals heterojunction (vdWhs). On the basis of using the angle-fixed method, this biosensor not only has good real-time performance but also detection sensitivity enhancement. The optical anisotropy characteristic of BP is used to widen the dynamic detection range of biosensors. The simulation results show that the maximum detection sensitivity of the proposed biosensor is 258.6 deg/RIU. Compared with the bare-Ag film structure biosensor, the detection sensitivity was enhanced by 209.2% by 2D vdWhs. The use of anisotropic 2D material BP can not only enhance the detection sensitivity but also widen the detection range. When the fixed incident angle is *θ* = 5 deg, a maximum dynamic detection range enhanced factor of 123.1% can be achieved, and a detection sensitivity of 185.2 deg/RIU in the corresponding interval can be obtained. The proposed biosensor in this study has potential broad application prospects in several fields, such as biological detection.

## 1. Introduction

Surface plasmon resonance (SPR) sensing technology is a high-sensitivity, real-time, and widely applicable analytical detection technology, which is increasingly being used in practical applications, especially in the field of biological detection technology [1,2,3,4].

The principle of SPR sensing technology is based on the SPR effect of noble metals [5]. The free electrons on the surface of noble metals undergo collective oscillation away from the lattice position under the action of the electromagnetic field. When the oscillation frequency of surface plasmon polaritons (SPPs) matches the frequency of the incident electromagnetic field, the SPR effect occurs [6,7]. SPR spectroscopy is highly sensitive to the changes in the refractive index of the nearby environment of the metal surface [8,9], and it can be roughly divided into three types based on spectral types: angular-interrogation, wavelength-interrogation, and phase-interrogation [10,11,12]. Among them, angular-interrogation is one of the earliest SPR detection techniques developed and applied due to its extremely high stability and accuracy [13]. Currently, most mainstream SPR biomolecule detection instruments are based on this technology [7,10,13]. Angular-interrogation uses monochromatic light. By changing the incident angle of the incident light, the intensity of the reflected light changes, and the incident angle corresponding to the minimum reflected light intensity is obtained, which is the SPR resonance angle [7,9]. The position of the SPR resonance angle is very sensitive to the refractive index near the detection interface, so the refractive index change on the surface of thin metal films can be detected by changing the resonance angle. Then, based on the linear relationship, the concentration or change in the content of the analyte can be obtained [9].

Since its inception, angular-interrogation SPR biosensing technology has reached remarkable achievements in both application expansion and detection performance improvement [14]. By surface modification and angular-interrogation detection techniques, specific target molecules can be detected [15]. At present, angular-interrogation SPR sensors have been widely used in many fields such as biochemical detection and so on [10,16,17]. In addition, significant achievements have been made in improving detection sensitivity through the hybridization effect between surface plasmas in multi-layer metal thin films or metal film/particle composite structures [18]. Moreover, due to the significant development of various new two-dimensional (2D) material research in recent years, some 2D materials with special optoelectronic properties have been used in the design of increasing SPR detection sensitivity [19,20,21,22]. Unlike bulk crystals, 2D materials such as graphene, transition metal dichalcogenides (TMDCs), and BP exhibit fascinating optical, chemical, and electronic properties, making them highly promising for designing novel plasmonic sensing devices [23,24,25]. A large number of research results have confirmed that effectively integrating 2D materials into traditional SPR sensors can result in unprecedented detection sensitivity enhancement [12,23,24,25,26]. This is mainly due to the significant electric field enhancement generated at the 2D atomic material metal interface, which is crucial for the development of SPR detection sensitivity. The first 2D material introduced for use was graphene, which was also one of the earliest 2D materials studied and prepared [25,26,27]. Its preparation process is mature, and its performance is stable, making it widely used. More importantly, due to the π–π interaction between the graphene surface and the tested biomolecules [12], graphene’s application in biological detection is becoming increasingly widespread. Secondly, black phosphorus, as a promising new type of 2D single-element material besides graphene, has received increasing attention in recent years. BP belongs to the VA family of layered 2D materials, and its unique optoelectronic properties make it a promising candidate for designing novel electronic, optoelectronic, and SPR devices [23,24]. Of particular importance is that BP is an optically anisotropic 2D material with different dielectric constants in three coordinate directions [12]. By utilizing this characteristic, tunable SPR sensors have been designed [12]. The disadvantages of black phosphorus are also obvious. Its chemical properties are relatively active and easy to react in the environment. Therefore, it is often stacked with other 2D materials to form heterojunctions. On the basis of ensuring its optical properties, it is covered with chemically stable 2D materials such as graphene [28,29]. This structure is increasingly being adopted and widely used in various directions.

Angular-interrogation SPR biosensing technology initially used a mechanical rotating device to adjust the incident angle, and then obtained the SPR reflectivity (intensity) at each angle, obtained the reflectivity curve, and then, obtained the position of the SPR resonance angle. By detecting the change in the SPR resonance angle, the refractive index change in the target detection substance was obtained. Although this method has stable performance and reliable results, it is limited by the fact that the process of adjusting the incident angle by the mechanical rotating device takes a long time and has poor real-time performance [30]. Therefore, it has obvious disadvantages in detecting with fast reaction speed. In order to meet the real-time detection requirements, the angle-fixed light incidence method has gradually been adopted [31]. After the incident light passes through the detection interface, the reflected light intensity is received by a charge-coupled device (CCD) or spectrometer to obtain the reflected spectrum, thereby obtaining the position of the SPR resonance angle. The continuous collection of reflectance spectra can effectively detect changes in the refractive index at the interface in real-time, thereby further obtaining the concentration or content of the target analyte. This method can quickly obtain reflection spectra and has good real-time detection performance. However, due to the limited range of incident light angles or CCD detection range, it is difficult to balance the sensitivity and dynamic detection range. To obtain a larger dynamic detection range, the detection sensitivity will decrease. Therefore, there is still a need for improvement in this method.

Overall, the development of angular-interrogation SPR sensors has shown very mature technical characteristics, with the advantages of high stability and reliable results. However, the inherent disadvantages of this approach have also limited the expansion of its application. Therefore, based on the black phosphorus (BP)/graphene 2D vdWhs, this work designed a biosensor with a maximum detection sensitivity of 258.6 deg/RIU. And by introducing the few-layer BP, the dynamic detection range was widened. When the fixed angle range was *θ* = 5 deg, a dynamic detection range of 1.330–1.363 was achieved, with a dynamic detection range increase of 123.1%, and a corresponding detection sensitivity of 185.2 deg/RIU was obtained. This design is expected to be widely applied in the field of biological detection, especially with its advantages of improving detection sensitivity and expanding dynamic detection range. It has significant technical advantages in the real-time detection of biological samples such as cells and tissues.

## 2. Materials and Methods

### 2.1. Architecture

**Figure 1:**
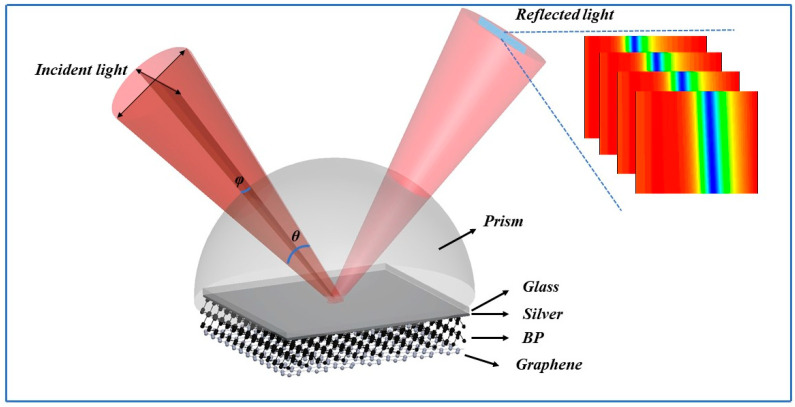
Schematic diagram of 2D vdWhs-enhanced SPR device. The reflectance spectrum image in the figure (top right), with pseudo color representing the intensity distribution of the reflected light, and blue to red indicating the strength of the reflected light intensity.

An angular-interrogation SPR biosensor composed of Ag film, few-layer BP, and monolayer-graphene is proposed, as shown in Figure 1. The commonly used Kretschmann model is used to excite SPR for the operation of biosensors [32]. The ambient temperature was set to be constantly 20 °C. This temperature condition aims to simulate the sensing performance of this SPR biosensor under a standard room temperature environment. A Ag-coated BK7 glass slide is closely attached to the edge of an equilateral prism (BK7) with the help of index-matching fluid, the side of the glass is tightly attached to the prism. BP layers are deposited on the surface of the Ag film. Therefore, monolayer graphene was deposited onto the BP layers. It has been confirmed that graphene can effectively enhance detection sensitivity and also efficiently adsorb aromatic biological molecules via the pi-stacking force [33]. In fact, there is another important reason for the use of graphene, which is the oxidizability of BP [12]. It may cause instability in the results by BP exposure to the sample environment. The use of graphene can effectively protect the material of BP, prevent direct contact with the environment, and effectively improve the stability and accuracy of the biosensor. A *p*-polarized light with a wavelength of 632.8 nm is employed to excite the SPR in the heterostructure (Ag/BP/graphene film). The incident light is focused by a lens and passes through the prism with a fixed angle range of *θ* = 5 deg, illuminating the bottom surface of the prism and exciting the SPR effect, and it reflects off the prism. The reflected light is recorded by an area array CCD for the real-time analysis of the changes in the refractive index. Therefore, the concentration of biological analytes in the sensing medium can be determined by monitoring the readout of the variations in the SPR signal.

### 2.2. Materials

The simulation of the performance of biosensors based on 2D vdWhs mainly involves five materials, including prisms, glass substrates, silver films, few-layer black phosphorus, monolayer graphene, and samples.

From top to bottom in Figure 1, the refractive index (n_BK7_) of the BK7 prism is 1.515 [34]. The thickness of the glass slide is set at 100 nm in our simulation model, and the material of the glass substrate is also BK7, with a refractive index of 1.515. The complex refractive index of Ag film at 632.8 nm is $n_{silver}=$ 0.056206 + 4.2776*i*, which was obtained by reference [35]. The calculation method for the refractive index of 2D BP is provided in the Supplementary Materials (Part B in Supporting Information) and comes from reference [12]. It should be noted that BP has optical anisotropy, and its refractive index is closely related to the angle between the incident light on the BP surface and the BP optical axis, which is represented by *φ*. Therefore, for incident light fixed at a certain angle range (θ = 5 deg in this work), SPR reflection spectra in different *φ* will exhibit different SPR resonance angles. It is precisely because of this optical characteristic that the dynamic detection range of biosensors is effectively expanded. The thickness of the BP layer $d_{BP}=L1\times 0.53\;\mathrm{nm}$, where *L1* is the number of BP layers, and the thickness of the monolayer BP is about 0.53 nm [12]. Under an excitation of 632.8 nm, the complex refractive index of graphene can be described by *n*_graphene_ = 3.0000 + 1.1487*i* [12]. The thickness of monolayer graphene is about 0.34 nm. The thickness of the graphene layer $d_{graphene}=L2\times 0.34\;\mathrm{nm}$, where *L2* is the number of graphene layers. Finally, the refractive index of the sample is given by the following relation (1) [12].

(1) $$n_{sample}=1.330+\Delta n_{bio}$$

where $\Delta n_{bio}$ is the tiny variation in the refractive index induced by the binding of targeted analytes. The materials and corresponding refractive index values used in the above simulation calculations are shown in Table 1.

### 2.3. Simulation Method

The SPR reflection spectrum used for biosensor detection is calculated using the transfer matrix method (TMM) and Fresnel equations based on an *N*-layer model in the Ag/BP/graphene sensing system (Part A in Supporting Information) [12]. Each layer is stacked together according to the order introduced above (as shown in Figure 1).

In this work, we determined the biosensor performance by employing four parameters (detection sensitivity (*S*), dynamic range enhancement factor (*E_W_*), detection sensitivity enhancement factor (*E_F_*), and figure of merit (*FOM*)). The detection sensitivity ($S_{Ag-VdWhs}$) of our proposed model is determined by Equation (2) [34]:

(2) $$S_{Ag-VdWhs}=\frac{\Delta \alpha }{\Delta n_{bio}}$$

where Δα is the differential SPR angle change for the *Ag-VdWhs* film. The dynamic range enhancement factor (*E_W_*) with *Ag-VdWhs* film vs. bare *Ag* thin film can be given as the following Equation (3):

(3) $$E_{W}=\frac{\Delta n_{bio-Ag}}{\Delta n_{bio-Ag-VdWhs}}\times 100\%$$

where $\Delta n_{bio-Ag-VdWhs}$ and $\Delta n_{bio-Ag}$ is the dynamic detection range for *Ag-VdWhs* film and the bare *Ag* thin film, respectively. In addition, the detection sensitivity enhancement factor (*E_F_*) with *Ag-VdWhs* film vs. bare *Ag* thin film can be given by Equation (4) [12]:

(4) EF=SAg−VdWhs SAg×100%=ΔαAg−VdWhs ΔnbioΔαAgΔnbio×100%=ΔαAg−VdWhsΔαAg×100%

where $\Delta \alpha_{Ag-VdWh}$ and $\Delta \alpha_{Ag}$ are the differential SPR angle changes for the *Ag-VdWhs* film and the bare *Ag* thin film, respectively. Another parameter, *FOM* is calculated. It is an important parameter to measure the sensor performance. In general, the greater the *FOM* value, the better the sensor performance. It is calculated by Formula (5) [36]:

(5) $$\;\;\;\;\;\;\;FOM=\frac{S_{VdWhs-Ag}}{FWHM}$$

where *FWHM* is full width at half maximum (*FWHM*).

## 3. Results and Discussion

**Figure 2:**
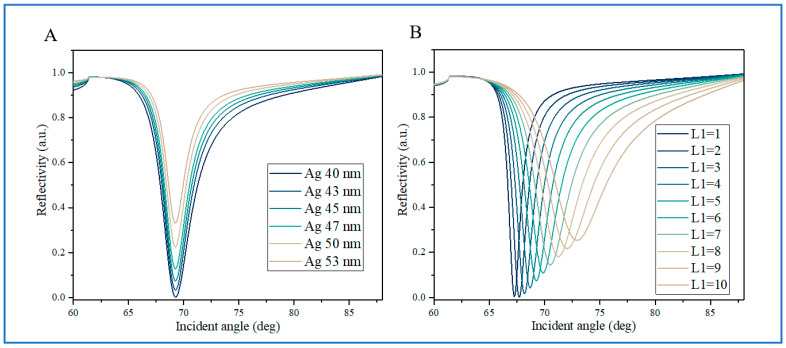
(**A**): The variation in reflectivity vs. the incident angle by varying different thicknesses of Ag film. The refractive index of the sample medium layer is set to 1.330 with an excitation wavelength of 632.8 nm. The layer number of the BP is five. (**B**): The variation in reflectivity vs. the incident angle by varying different BP layer numbers. The refractive index of the sample medium is set to 1.330 with an excitation wavelength of 632.8 nm. The thickness of the Ag film is 45 nm.

The angular-interrogation SPR sensor is based on the principle that the SPR resonance angle is sensitive to the refractive index at the detection interface. The function of reflectivity (*R*_p_) and incident angle (*θ*) is called the SPR reflection curve or SPR reflection spectrum, where the SPR resonance angle corresponds to the incident light angle at the minimum reflectivity of the curve. The change in the SPR resonance angle position caused by changes in the surface refractive index is used to detect the surface refractive index. In the calculation, the refractive index of the measured medium is first fixed at 1.330, corresponding to the refractive index of deionized water, to analyze the structural parameters of the biosensor. As shown in Figure 2, the spectrum variation of the proposed SPR sensor was systematically studied by changing the thickness of the Ag film and BP. Firstly, the influence of the Ag film thickness on the SPR reflectance spectra was investigated (Figure 2A). The thickness of the BP was fixed at five layers, and the thickness of the graphene was fixed at a monolayer. The Ag film thickness varied from 40 nm to 53 nm. The results showed that the change in the SPR resonance angle was not significant, but the minimum reflectance increased with the increase in Ag film thickness. The minimum reflectance of the Ag film with a thickness of 53 nm was higher than that of the Ag film with a thickness of less than 40 nm. This was mainly due to the increase in electron energy loss caused by the increase in Ag film thickness. The size of the minimum reflectance had a certain impact on the *FOM* of the SPR spectra. Generally, a smaller minimum reflectance often had a higher *FOM*.

Then, for the investigation of the effect of the BP thickness on the SPR spectra, the simulation results show that for fixed-thickness Ag films and graphene layers, the SPR resonance angle (Figure 2B) first shows a significant red shift with increasing BP thickness. At the same time, the SPR reflectance curve also widens with the thickness increase in the BP layer (Figure 2B), which may be due to the increased thickness of the BP layer causing an increase in energy loss. In addition, it was found that the minimum reflectivity is closely related to the thickness of the BP on the Ag film (Figure 2B), and the minimum reflectivity gradually increases with the increase in BP thickness. In fact, the energy absorbed solely by the Ag film is not sufficient to generate strong SPR excitation [12]. By utilizing the light absorption caused by the BP layer, the absorption energy of the Ag/BP interface can be effectively increased, resulting in strong SPR excitation.

**Figure 3:**
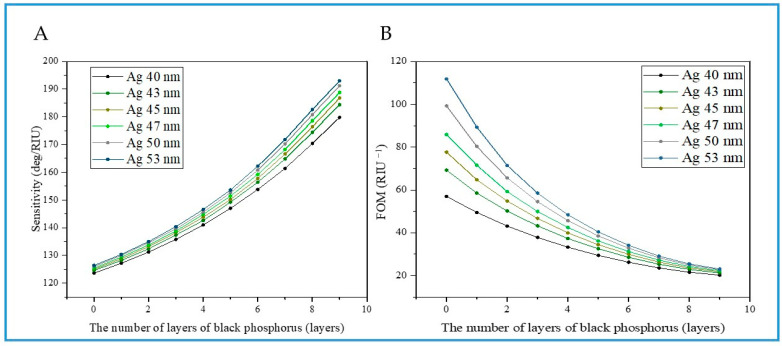
(**A**): The variation in detection sensitivity *S* vs. the different BP layer numbers by varying different thicknesses of Ag film. The refractive index of the sample medium is set to 1.330–1.380 with an excitation wavelength at 632.8 nm. (**B**): The variation in *FOM* vs. the different BP layer numbers by varying different thicknesses of Ag film. The refractive index of the sample medium is set to 1.330–1.380 with an excitation wavelength of 632.8 nm.

For angular-interrogation SPR biosensors, detection sensitivity *S* and *FOM* are the two most important parameter indicators. Therefore, we investigated the effects of Ag film and BP thickness changes on detection sensitivity and *FOM* (as shown in Figure 3). The simulation results show that the detection sensitivity increases with the increase in BP thickness when the Ag film thickness is 40 nm, 43 nm, 45 nm, 47 nm, 50 nm, and 53 nm within the range of 1.330–1.380 for the refractive index change in the sample (Figure 3A). And as the thickness of the Ag film increases, the detection sensitivity also gradually increases, with a maximum detection sensitivity of 193 deg/RIU. At this time, the sensor structural parameters are Ag 53 nm, 9-layer BP, and monolayer graphene. In fact, although S increases with the thickness of BP, at the same time, the *FOM* of the sensor shows the opposite trend (Figure 3B), mainly due to the stronger light absorption brought by the increase in BP thickness, and the gradually increasing *FWHM*. However, the difference is that the size of *FOM* increases with the increase in Ag film thickness, which further indicates that the increase in Ag film thickness brings stronger SPR excitation. The maximum *FOM* value is 111.9 RIU^−1^, obtained under the conditions of Ag 53 nm, zero-layer BP, and monolayer graphene (see Table 1). The above simulation results indicate that *FOM* and *S* have opposite trends in change. Therefore, in specific biological detection applications, it is necessary to balance the detection sensitivity *S* and *FOM* and comprehensively consider selecting the appropriate sensor structure in order to achieve optimal detection performance.

**Figure 4:**
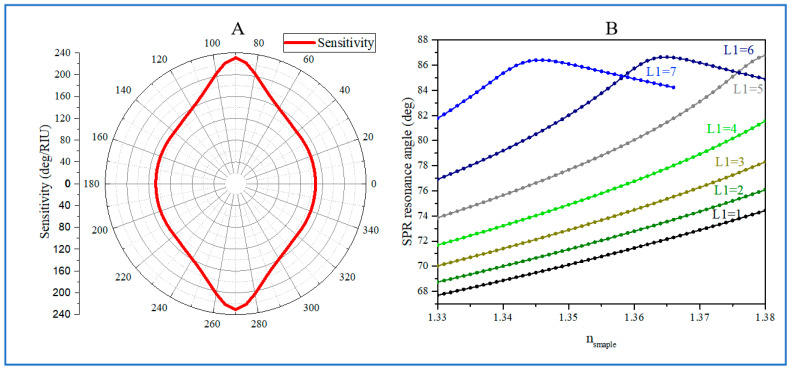
(**A**): The variation in detection sensitivity *S* vs. the angle *φ*. The refractive index of the sample medium is set to 1.330–1.380 with an excitation wavelength of 632.8 nm. (**B**): The variation in the SPR resonance angle *θ* vs. the refractive index of the sample by varying different BP layer numbers. The thickness of the Ag film is fixed at 53 nm with an excitation wavelength of 632.8 nm.

The detection sensitivity and *FOM* of Ag/BP/graphene structure biosensors are closely related not only to the thickness of the Ag film and BP but also to the anisotropic optical properties of BP. The detection sensitivity and *FOM* of biosensors are also related to the angle *φ*, as shown in Figure 4A. We summarized the detection sensitivity *S* of biosensors corresponding to the angle *φ*. The data show that the biosensor has maximum detection sensitivity when *φ* = 90 deg and *φ* = 270 deg, while *φ* = 0 deg and *φ* = 180 deg have minimum detection sensitivity.

Next, we investigated the detection sensitivity *S* of the biosensor with a *φ* of 90 deg, as shown in Figure 4B. In the case of a 53 nm Ag film and monolayer graphene, with a 1–7-layer thickness BP, the SPR resonance angle position varied with the refractive index Δ*n*. As a result, it was found that when the thickness of BP was greater than five layers, the SPR resonance angle had a significant red shift and gradually approached 90 deg, resulting in overlapping reflection angles (as shown in the two curves L1 = 6 and L1 = 7 in Figure 4B). This structure obviously cannot be used for biological detection. It had the same pattern under the change in Ag thickness. Therefore, when *φ* = 90 deg, the maximum thickness of BP can be stacked up to five layers within the range of 40–53 nm for Ag film. Table 2 lists the corresponding detection sensitivity and FOM for Ag film thicknesses of 40 nm, 43 nm, 45 nm, 47 nm, 50 nm, and 53 nm at *φ* = 90 deg. It can be found that as the Ag film thickness increases, the detection sensitivity gradually increases, reaching its maximum at 50 nm and then slightly decreasing. The *FOM* also shows the same trend. However, for the bare Ag film structure sensor, it has the smallest detection sensitivity but the largest *FOM*. By comparison, the maximum detection sensitivity reached 258.6 deg/RIU, which was obtained in a 50 nm Ag film, five-layer BP, and monolayer graphene structure. Compared with the bare Ag film structure, the detection sensitivity increase factor *E_F_* value was 209.2%. At the same time, this structure also had the largest *FOM* value of 44 RIU^−1^. See Table 2.

Compared with other SPR biosensor structures based on the BP/2D material heterojunctions shown in Table 3, the sensitivity of our proposed hybrid structure is at a normal level, which elucidates the contribution of BP to the performance of the SPR biosensors. Most of these sensor systems exhibit acceptable sensitivity in the range of 200 deg/RIU to 400 deg/RIU. It should be noted that this work mainly focuses on designing an SPR biosensor with a simple structure that balances detection sensitivity and dynamic detection range. Therefore, a moderate level of detection sensitivity is acceptable.

The anisotropic 2D vdWhs BP/graphene structure not only significantly improves detection sensitivity but also has the important advantage of utilizing anisotropic 2D BP, shown in Table 4, which can significantly enhance the dynamic detection range. In order to better evaluate the dynamic detection range of the proposed Ag/BP/graphene structure SPR biosensor, the changes in dynamic detection range were tested within the fixed incident light angle range *θ* = 5 deg. Obviously, the angle range of the angle *φ* is also 5 degrees.

**Figure 5:**
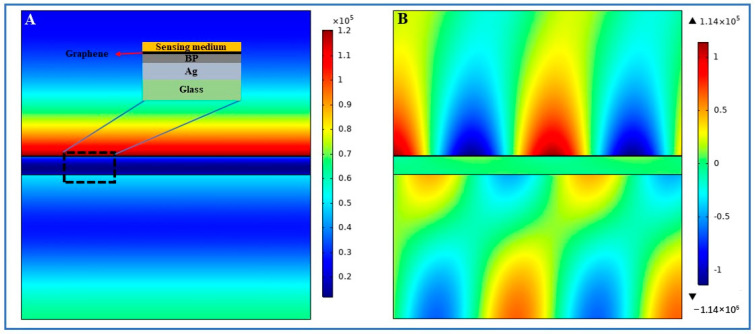
(**A**): The electric field intensity distribution; (**B**) the y-component distribution of the electric field. The thickness of the Ag film is 50 nm, and the thickness of the BP and graphene is five-layer and monolayer, respectively, with an excitation wavelength of 632.8 nm.

Next, we employed finite element analysis to simulate the electric field distribution when the SPR resonance angle was 69.27 deg to determine the strongly enhanced electric field generated by hybrid architectures comprising 5-layer BP, monolayer graphene, and 50 nm Ag film. Figure 5 shows an enhanced electric field that was significantly excited at the sensing interface (Figure 5A shows the electric field intensity distribution, and Figure 5B shows the y-component distribution of the electric field intensity). It can be concluded that this Ag/BP/graphene structure achieved a maximum electric field enhancement of 1.2 × 10^5^ times at the detection interface.

**Figure 6:**
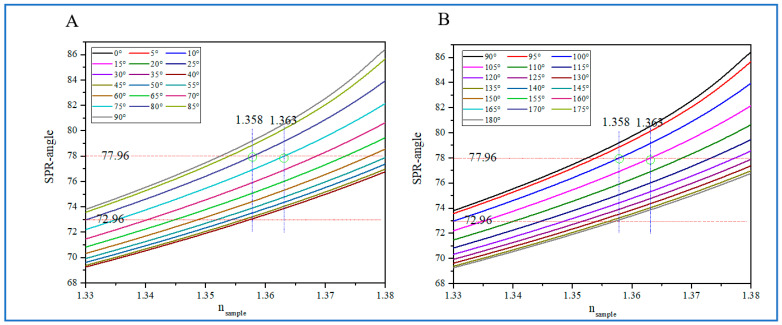
(**A**): The variation in the SPR resonance angle within *φ* for the range of 0–90 deg. (**B**) The variation in the SPR resonance angle within *φ* for the range of 90–180 deg. The thickness of the Ag film is 45 nm, and the thickness of the BP and graphene is five-layer and monolayer, respectively, with an excitation wavelength of 632.8 nm.

In order to better increase the dynamic detection range, the influence of the variation range of *φ* on the dynamic detection range is first examined, as shown in Figure 6 for the 45 nm Ag film, five-layer BP, and monolayer graphene structure. The law of the SPR resonance angle changing with the refractive index in different ranges of *φ* is investigated. Figure 6A represents the variation in the SPR resonance angle within *φ* in the range of 0–90 degrees, while Figure 6B represents the variation in the SPR resonance angle within *φ* in the range of 90–180 degrees. It was found that the biosensor has the maximum dynamic detection range when *φ* is within the range of *φ* = 75–80 deg and *φ* = 100–105 deg. And under the variation in Ag and BP thickness, the same pattern is observed. Therefore, in practical applications, the 75–80 deg and 100–105 deg angle *φ* ranges are the best choices. Therefore, the next analysis will select the 75–80 deg angle *φ* range.

**Figure 7:**
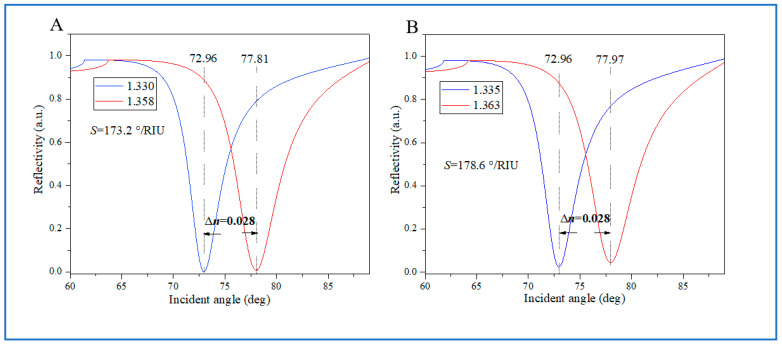
(**A**): The variation in the reflectivity vs. the incident angle by the refractive index of the sample is 1.330 and 1.358 in *φ* = 80 deg. (**B**): The variation in the reflectivity vs. the incident angle by the refractive index of the sample is 1.335 and 1.363 in *φ* = 75 deg. With an excitation wavelength of 632.8 nm. The thickness of the Ag film is 45 nm, and the thickness of the BP and graphene is five-layer and monolayer, respectively.

Next, we investigated the effect of the anisotropy of BP on the dynamic detection range in the case of Ag thickness for 45 nm, five-layer BP, and a monolayer graphene structure. As shown in Figure 7A, when the angle *φ* = 80 deg, the refractive index of the sample is 1.330, corresponding to an SPR resonance angle of 72.96 deg. Due to the limitation of the incident light angle, the refractive index of the sample to be detected changes from 1.330 to 1.358 within the range of 72.96 deg + *θ*, with a Δ*n* of 0.028 and a detection sensitivity of 173.2 deg/RIU. At the same time, the angle *φ* brought from the optical anisotropy of BP has a range of 5 degrees too. Therefore, the refractive index of BP has a significant change within this angle range, thereby increasing the dynamic detection range. As shown in Figure 7B, when *φ* = 75 deg, the refractive index detection range is 1.335–1.363 within the angle range 72.96 deg + *θ*, Δ*n* is 0.033, and the detection sensitivity is 178.6 deg/RIU. Therefore, the overall dynamic detection range of the biosensor with the structure of Ag thickness for 45 nm, five-layer BP, and monolayer graphene is 1.330–1.363, and the corresponding detection sensitivity is a minimum of 173.2 deg/RIU. As a result, a significant increase in the dynamic detection range has been achieved, with Δ*n* increasing from 0.028 to 0.033. An increase of 0.005 and an increase factor *E_W_* value of 117.9% were obtained.

**Figure 8:**
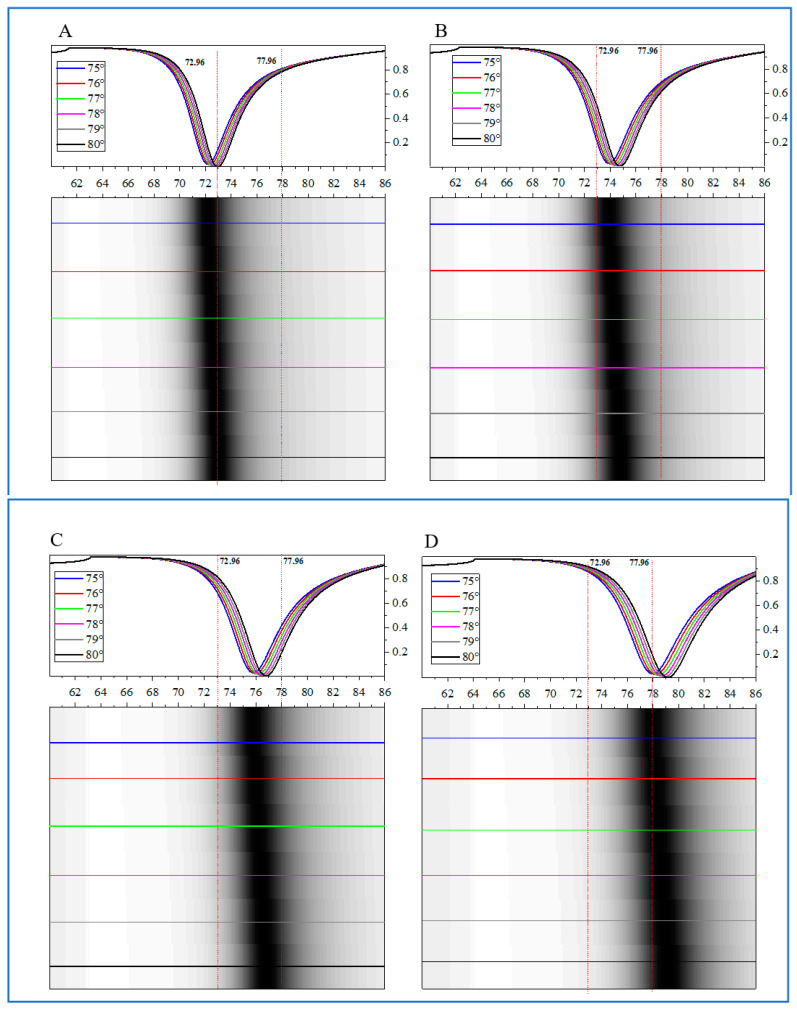
The SPR spectra and light intensity distribution as the refractive index of the detection medium of the proposed biosensors: (**A**), 1.330; (**B**), 1.341; (**C**), 1.352; (**D**), 1.363. The thickness of the Ag film is 45 nm, and the thickness of the BP and graphene is five-layer and monolayer, respectively, with an excitation wavelength of 632.8 nm.

At the same time, we investigated SPR spectra and reflected light intensity distribution as the refractive index of the detection medium indices of 1.330, 1.341, 1.352, and 1.363 of the proposed biosensors, as shown in Figure 8A–D. Due to the anisotropic optical properties of BP, the angle *φ* has an angle range of five degrees, corresponding to 75–80 deg as shown in Figure 8A–D. The SPR spectra are shown in the upper figures, and the corresponding reflected light intensity distribution is shown in the lower figures. The simulation results show that when the refractive index is 1.330, only the spectrum of *φ* = 80 deg can be recognized in the image within the detection angle range of 72.96–77.96 deg. At this point, as the minimum detection boundary of the biosensor. As the refractive index continues to increase, the SPR resonance angle gradually shows a red shift. At a refractive index of 1.341, one can observe the SPR resonance angle in all *φ* corresponding to this refractive index until the refractive index reaches 1.363 (Figure 8D). At this point, only when *φ* = 75 deg can the SPR resonance angle corresponding to this refractive index be observed. Therefore, this is the maximum dynamic detection boundary of the biosensor. Then, we can calculate that the proposed biosensor has expanded its dynamic detection range from 1.330–1.358 to 1.330–1.363, with an enhanced factor *E_W_* value of 117.9%.

Table 4 lists the maximum dynamic detection range and corresponding *FOM* values for Ag film thicknesses of 40 nm, 43 nm, 45 nm, 47 nm, 50 nm, and 53 nm with the same thickness of BP and graphene. By comparison, it can be concluded that the Ag film with a thickness of 53 nm, five layers of BP, and a monolayer graphene structure has a maximum dynamic detection range of 1.330–1.362, and the dynamic range increase factor *E_W_* is 123.1%. And it has a relative maximum detection sensitivity of 185.2 deg/RIU, with a corresponding *FOM* of 40.4 RIU^−1^.

## 4. Conclusions

To conclude, in this work, we proposed an angular-interrogation biosensor based on BP/graphene anisotropic 2D vdWhs. Firstly, this biosensor adopts the fixed-angle light incidence method and also has the advantage of good real-time performance. Secondly, with the use of 2D vdWhs, the proposed biosensor achieved a maximum detection sensitivity of 258.6 deg/RIU, which is 209.2% enhanced compared to bare-Ag thin-film architecture. More importantly, due to the optical anisotropy of the 2D material BP, this biosensor not only ensures an enhancement in detection sensitivity but also achieves an improvement in the dynamic detection range, achieving a 123.1% enhancement and with a maximum detection sensitivity of 185.2 deg/RIU. This design is convenient and easy to implement and is expected to have broad application prospects in fields such as biosensitivity detection and biomedical applications.

## Figures and Tables

**Table 1:** The refractive index of different materials at 632.8 nm.

Layer	Materials	Refractive Index at = 632.8 nm		Reference
		Real Part (*n*)	Imaginary Part (*k*)	
Layer I	BK7 prism	1.515	---	[34]
Layer II	Glass substrate	1.515	---	[34]
Layer III	Silver	0.056206	4.2776*i*	[35]
Layer IV	BP *	2.77	0.507i	
		2.716	0.033	[12]
		6.1	---	
Layer V	Graphene	3	1.1487	[12]
Layer VI	Sensing medium	1.330 + Δ*n*_bio_	----	[12]

* The refractive index of BP material is the value when *φ* is 0 deg, and its refractive index varies with the angle *φ*, as shown in reference [12].

**Table 2:** The optimized values of the Ag film thickness, the BP layer numbers, the detection sensitivity *S,* and the *FOM*.

Ag Thickness (nm)	BP Thickness (L1)	Graphene Thickness (L2)	Sensitivity (deg/RIU) (φ = 90, Δ*n_bio_* = 0.05)	*FOM* (RIU^−1^)
40	0	0	123.6	57.0
40	5	1	231.0	35.2
43	5	1	245.0	39.4
45	5	1	252.2	41.8
47	5	1	256.4	43.3
50	5	1	258.6	44.0
53	5	1	257.8	43.2

**Table 3:** Several recent SPR biosensors based on BP: biosensor structure, incident wavelength (nm), sensitivity (deg/RIU), and corresponding reference.

Biosensor Structure	Incident Wavelength (nm)	Sensitivity (deg/RIU)	Reference
Bimetallic/BP/WSe_2_	633	237.2	23
Ag/BP-MXene-BP	633	295.67	24
Ag/graphene/BP	633	236.64	25
Ag/BP/Cu-Pt/graphene	633	351.66	26
Cu/Ni/Graphene/BP	633	410	27
Au/Ag/GaN/BP	633	440	28
Ag/BiFeO_3_/BP	633	356.19	29
Ag/BP/graphene	632.8	258.6	This work

**Table 4:** The optimized values of the Ag film thickness, the number of BP layers, the dynamic detection range, the sensitivity, and the *FOM*.

Ag Thickness (nm)	BP Thickness (Number of Layers)	Dynamic Detection Range (∆θ = 5 deg, φ = 80 deg)	Dynamic Detection Range (∆θ = 5 deg, φ = 75 deg)	Sensitivity (deg/RIU) (∆θ = 5 deg, φ = 75 deg)	*FOM* (RIU^−1^)
40	5	1.330–1.359	1.335–1.364	172.4	27.7
43	5	1.330–1.358	1.335–1.363	176.1	31.1
45	5	1.330–1.358	1.335–1.363	178.6	33.2
47	5	1.330–1.357	1.335–1.362	180.0	35.4
50	5	1.330–1.357	1.335–1.362	182.6	38.1
53	5	1.330–1.356	1.335–1.362	185.2	40.4

## Data Availability

The data presented in this study are available on request from the corresponding author.

## Supplementary Materials

The following supporting information can be downloaded at: https://www.mdpi.com/article/10.3390/bios14120601/s1, Part A: Transfer matrix method (TMM) for reflection of multilayer films and Part B: Refractive Index calculation of anisotropic black phosphorus (BP) Figure S1 Schematic diagram of coordinate transformation of dielectric axis (x, y, z), crystal axis (a, b, c), and laboratory coordinate system (x″, y″, z″) [5,7,12].
