# Supplementary material for: Widening of Dynamic Detection Range in Real-Time Angular-Interrogation Surface Plasmon Resonance Biosensor Based on Anisotropic Van Der Waals Heterojunction
Source: Biosensors (Basel). 2024 Dec 8;14(12):601. doi: 10.3390/bios14120601 (PMC11674669; doi:10.3390/bios14120601)
Supplement: Supplementary file 1 [file biosensors-14-00601-s001.zip › biosensors-3291962-supplementary.pdf]

Supporting Information

Widening of Dynamic Detection Range in Real-Time Angular-Interrogation Surface Plasmon Resonance Biosensor Based on Anisotropic Van Der Waals Heterojunction

Xiantong Yu *, Jing Ouyang, Zhao Li, Chaojun Shi, Longfei Wang, Jun Zhou and Min Chang

Key Laboratory of Optical Technology and Instrument for Medicine, Ministry of Education, College of Optical-Electrical and Computer Engineering, University of Shanghai for Science and Technology, Shanghai 200093, China

* Correspondence: xtyu@usst.edu.cn

Part A: Transfer matrix method（TMM）for reflection of multilayer films:

The SPR reflection spectrum used for biosensor detection in this work is calculated using the transfer matrix method (TMM) and Fresnel equations based on an N-layer model in Ag/BP/graphene sensing system [1–2].

Reflection coefficient, r_p, can be described by Fresnel equation and Snell's law:

$$r_p = \frac{E_{rp}}{E_{ip}} = \frac{n_t \cos\theta_i - n_i \cos\theta_t}{n_t \cos\theta_i + n_i \cos\theta_t}, \quad \text{(S1a)}$$

$$n_i \sin\theta_i = n_t \sin\theta_t, \quad \text{(S1b)}$$

n_i and n_t are the refractive indexes of the two media respectively.

For multi-layer case, the transfer matrix can be described as follows:

$$M = \prod_{k=2}^{N-1} M_k = \begin{bmatrix} M_{11} & M_{12} \\ M_{21} & M_{22} \end{bmatrix}, \quad \text{(S2a)}$$

$$M_k = \begin{bmatrix} \cos\beta_k & -i\dfrac{\sin\theta_k}{q_k} \\ -iq_k \sin\beta_k & \cos\beta_k \end{bmatrix}, \quad \text{(S2b)}$$

$$q_k = \frac{\left(\varepsilon_k - n_{SF11}^2 \sin^2\theta_1\right)^{1/2}}{\varepsilon_k}, \quad \text{(S2c)}$$

$$\beta_k = \frac{2\pi n_k cos\theta_k (Z_k - Z_{k-1})}{\lambda} = \frac{\pi d_k}{2}(\varepsilon_k - n_1^2 sin^2\theta_1)^{1/2}, \quad \text{(S2d)}$$

n_k is Refractive index of the k-th layer，θ_1 is the angle of incidence of the first layer，ε_k and d_k are the dielectric function and thickness of the k-th layer respectively.

The first boundary of the tangential field is defined as Z_1＝0，last boundary Z_n＝1, then the transfer matrix can be described:

$$\begin{bmatrix} U_1 \\ V_1 \end{bmatrix} = M \begin{bmatrix} U_{N-1} \\ V_{N-1} \end{bmatrix}, \quad \text{(S3)}$$

Where U is the tangential component of the interface electric field and V is the tangential component of the magnetic field. Therefore, for the n-th layer, p-polarized light, the reflection coefficient is，

$$r_p = \frac{(M_{11} + M_{12}q_N)q_1 - (M_{21} + M_{22}q_N)}{(M_{11} + M_{12}q_N)q_1 + (M_{21} + M_{22}q_N)}, \tag{S4}$$

And the reflection coefficient can be described:

$$R_P = |r_p|^2, \tag{S5}$$

Equation (S4)，(S5) is also applies to s-polarized light, and the parameters,

$$q_k = \left(\varepsilon_k - n_{SF11}^2 \sin^2\theta_1\right)^{1/2}. \tag{S6}$$

Part B: Refractive Index calculation of anisotropic black phosphorus (BP):

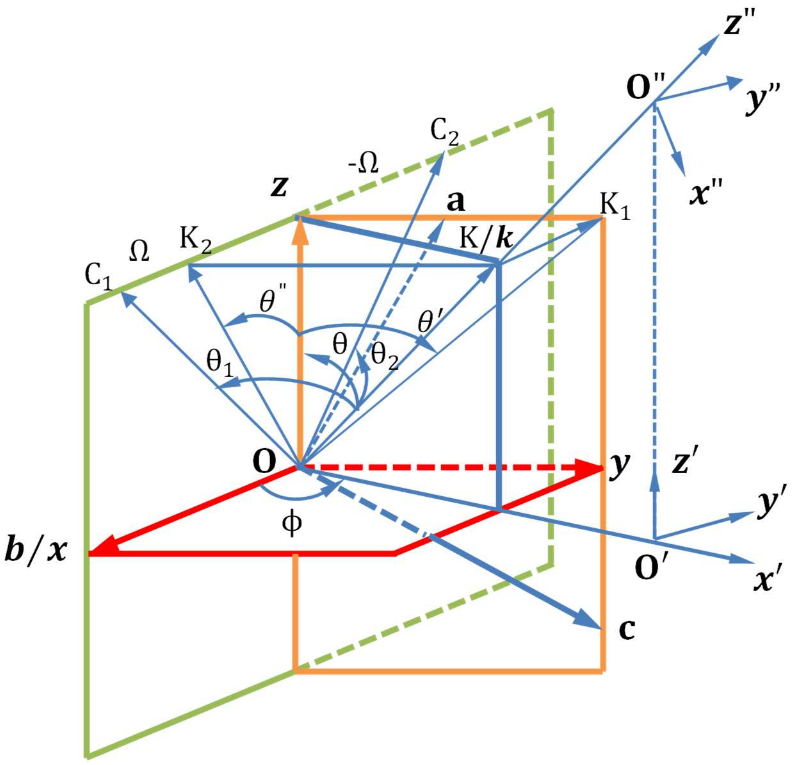

Figure S1. Schematic diagram of coordinate transformation of dielectric axis (x, y, z), crystal axis (a, b, c), and laboratory coordinate system (x '', y '', z '') [3].

As an optically anisotropic two-dimensional materials, the complex refractive index of the BP layer in changes with the angle of incident light, which has a significant impact on the performance of SPR biosensors based on BP/graphene van der Waals heterojunctions. In this work, we use coordinate transformation to calculate the complex refractive index of anisotropic BP [3]:

As shown in Figure S1, according to the Fresnel equation, the dielectric constant components in various directions can be obtained in the dielectric axis system (x， y， z), but the crystal parameters are measured in the crystal principal axis coordinates (a， b， c). In biaxial crystals, these two coordinate systems are not parallel. In order to calculate the relationship between the refractive index and angle of biaxial crystals, first, rotate the dielectric main axis coordinate xyz along the z-axis by an angle of φ to obtain the coordinate axis $x'y'z'$. Then, rotate the coordinate $x'y'z'$ along the y-axis by an angle of θ to obtain the laboratory coordinate axis $x''y''z''$ (as shown in Figure S1). Here, θ is the polar angle relative to the dielectric axis z in the $x'z'$ plane, and φ is the azimuth angle relative to the

dielectric axis x in the *xy* plane. The two crystal optical axes (C_1, C_2) are symmetrically located on either side of the z-axis, and the angles between them are Ω and - Ω, respectively. θ_1 and θ_2 are the angles between the wave vector ***k*** and the two optical axes C_1 and C_2, respectively. θ ^ 'and θ ^' 'are the angles between the projection of the wave vector ***k*** on the *yz* and *xz* planes and the *z*-axis, respectively. The optical axis angle is:

$$\Omega = \arcsin\left(\frac{n_z}{n_y}\sqrt{\frac{n_y^2-n_x^2}{n_z^2-n_x^2}}\right), \tag{S7}$$

here n_x, n_y,and n_z are the refractive indices of the *x, y* and *z* components, respectively.

The Fresnel equation states that the refractive index of two orthogonal polarized lights can be expressed as:

$$\frac{1}{n_{1,2}^2} = \frac{\cos^2\left(\frac{\theta_1 \pm \theta_2}{2}\right)}{n_x^2} + \frac{\sin^2\left(\frac{\theta_1 \pm \theta_2}{2}\right)}{n_z^2}。 \tag{S8a}$$

$$n_1 = n_p = \frac{n_x n_z}{\sqrt{n_x^2 \sin^2\left(\frac{\theta_1+\theta_2}{2}\right) + n_z^2\cos^2\left(\frac{\theta_1+\theta_2}{2}\right)}} \tag{S8b}$$

$$n_2 = n_s = \frac{n_x n_z}{\sqrt{n_x^2 \sin^2\left(\frac{\theta_1-\theta_2}{2}\right) + n_z^2\cos^2\left(\frac{\theta_1-\theta_2}{2}\right)}}。 \tag{S8c}$$

In the tetrahedrons O-KK_2C_1 and O-KK_2C_2,

$$\cos\theta_1 = \cos\angle K_2OK \bullet \cos(\angle C_1OK_2) = \frac{\cos\theta \bullet \cos(\Omega-\theta'')}{\cos\theta''} \tag{S9a}$$

$$\cos\theta_2 = \cos\angle K_2OK \bullet \cos(\angle C_2OK_2) = \frac{\cos\theta \bullet \cos(\Omega+\theta'')}{\cos\theta''} \tag{S9b}$$

$$0^\circ \le \theta < 90^\circ,\ 0^\circ \le \varphi \le 90^\circ;\ \theta'' = ar\tan(\tan\theta \bullet \cos\varphi), \tag{S9c}$$

$$90^\circ < \theta \le 180^\circ,\ 0^\circ \le \varphi \le 90^\circ;\ \theta'' = -ar\tan(\tan\theta \bullet \cos\varphi)。 \tag{S9d}$$

Equations (S8) and (S9) provide the conversion between the dielectric coordinate system and the laboratory coordinate system. Once the parameters (*Ω*, θ and φ) are determined, θ_1 and θ_2 can be determined and the refractive index of orthogonal polarized light can be obtained. In special circumstances, Ω=0, the material becomes a uniaxial crystal, therefore,

$$n_o = n_x,$$

$$\frac{1}{n_e^2} = \frac{\cos^2\theta}{n_x^2} + \frac{\sin^2\theta}{n_z^2}。 \tag{S10}$$

The refractive index of orthogonally polarized light (n_p, n_s) can be obtained from equation (S10).

The refractive indices of two orthogonal polarized lights determined based on coordinate transformation can correspond to the TM and TE excitation modes.

[1] Schasfoort, R.B.M.; Tudos, A.J. Handbook of Surface Plasmon Resonance; The Royal Society of Chemistry: Cambridge, UK, **2008**; pp. 1–13.

[2] Maier, S.A. Plasmonics: Fundamentals and Applications; Springer: New York, NY, USA, **2007**; pp. 49–74.

[3] Yuan, Y.F.; Yu, X.T.; Ouyang, Q.L.; Shao, Y.H.; Song, J.; Qu, J.L.; Yong, K.T. Highly anisotropic black phosphorous graphene hybrid architecture for ultrasensitive plasmonic biosensing: Theoretical insight. 2D Mater. **2018**, *5*, 025015.
